# One more step toward treatment of PARP inhibitor-resistant ovarian cancers

**DOI:** 10.18632/oncotarget.28545

**Published:** 2023-12-22

**Authors:** Upasana Ray, Prabhu Thirusangu, Viji Shridhar

**Keywords:** ovarian cancer, PARP inhibitors, PG545, DNA damage, cell death

The current scenario presents a stark reality: over 80% of ovarian cancer cases experience recurrence, resulting in roughly 12,000 annual deaths in the United States [1]. While targeted therapies like poly (ADP-ribose) polymerase inhibitors (PARPis) have received FDA approval for both initial and recurrent treatments, extending median progression-free survival for individuals with homologous recombination repair (HRR) deficiency, the emergence of PARPi resistance remains a common challenge among patients [2]. Consequently, addressing resistance to PARPi treatment in ovarian cancer has become a pressing therapeutic dilemma, necessitating innovative strategies.

In response to this unmet need, our current study has unveiled promising findings related to the Pixatimod (PG545) drug, a sulfated small molecule compound. Engineered with a core structure mimicking heparan sulfate, this compound targets heparanase and heparin binding growth factor (HB-GF) signaling [3]. Previous research has demonstrated PG545’s potential by enhancing chemotherapy responses through the inhibition of growth factor-mediated signaling in ovarian cancer and exhibiting anticancer activity in various preclinical models of breast, prostate, and lung cancer [4–7]. Our present study has revealed a previously unknown effect of PG545 in ovarian cancer cells, inducing DNA damage. The investigation unveiled that PG545 induces both single- and double-strand breaks in DNA while also promoting the autophagic degradation of RAD51, a critical DNA repair protein, thereby impeding the homologous recombination repair (HRR) pathway in cancer cells.

Beyond its canonical role in growth factor signaling, heparan sulfate proteoglycans (HSPGs) are also involved in the endocytosis of specific molecules [8]. We discovered that PG545 thwarts the internalization of the DEK oncoprotein within cancer cells. DEK is known to be upregulated in many cancers, playing a pivotal role in the DNA repair pathway, acting as a chemoattractant for inflammatory cells, and regulating inflammation in the tumor microenvironment [9–12]. By disrupting HSPG-mediated endocytosis, on which DEK relies for internalization, PG545 induces the loss of nuclear DEK and hinders the DNA repair pathway. Our further studies revealed that PG545 synergizes with PARPis in ovarian cancer cell lines *in vitro*. This synergy was observed even in cell lines resistant to PARPi monotherapy. Encouragingly, this interaction was reflected in 55% of primary cultures derived from patient ascites samples in an *ex vivo* model. Moreover, the combination of PG545 and the PARPi rucaparib reduced tumor burden in mice implanted with HRR-proficient OVCAR5 cell line xenografts. This trend was also evident in an *in vivo* setting using an immunocompetent syngeneic ID8F3 ovarian ascites xenograft model, illustrating the combination’s efficacy in diverse contexts.

**Figure 1:**
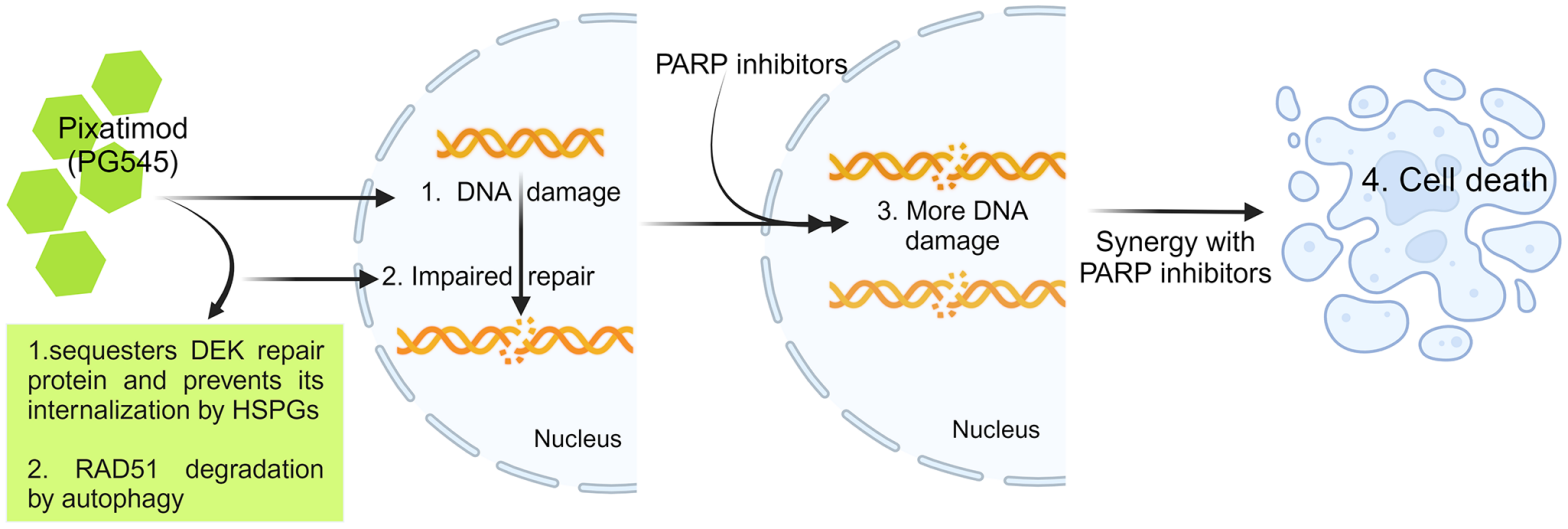
Representation of the work.

The study marks a breakthrough in understanding PG545’s role in initiating the DNA damage pathway in ovarian cancer cells (Figure 1). This novel therapeutic strategy, leveraging PG545 to sensitize ovarian cancer cells to PARPi treatment, carries significant implications. Importantly, the observed synergy is not solely dependent on HR deficiency, as PG545/PARPi synergy was evident in HR-deficient and PARPi-resistant lines. PG545’s ability to downregulate RAD51 already mentioned before in HR-proficient cell lines also contributes to PARPi sensitization, an area ripe for further preclinical exploration. This study suggests that PG545 may hold potential for addressing a specific subset of ovarian cancers characterized by resistance to PARPis, which are associated with unfavorable outcomes.

Therefore, we advocate the need for further *ex vivo* research involving ascites samples obtained from patients. These investigations are essential to establish connections between treatment responses and clinical characteristics or mutational profiles. Furthermore, incorporating patient- derived xenograft models in future studies will provide a more extensive assessment of the effectiveness of combination treatments, particularly in preclinical models that exhibit resistance to current therapies. This initiative is geared towards identifying additional biomarkers that can aid in selecting the most appropriate treatments for ovarian cancer.
